# Application of Wearable Inertial Sensors and A New Test Battery for Distinguishing Retrospective Fallers from Non-fallers among Community-dwelling Older People

**DOI:** 10.1038/s41598-018-34671-6

**Published:** 2018-11-05

**Authors:** Hai Qiu, Rana Zia Ur Rehman, Xiaoqun Yu, Shuping Xiong

**Affiliations:** Human Factors and Ergonomics Laboratory, Department of Industrial & Systems Engineering, Korea Advanced Institute of Science and Technology (KAIST), Daejeon, South Korea

## Abstract

Considering the challenge of population ageing and the substantial health problem among the elderly population from falls, the purpose of this study was to verify whether it is possible to distinguish accurately between older fallers and non-fallers, based on data from wearable inertial sensors collected during a specially designed test battery. A comprehensive but practical test battery using 5 wearable inertial sensors for multifactorial fall risk assessment was designed. This was followed by an experimental study on 196 community-dwelling Korean older women, categorized as fallers (N_1_ = 82) and non-fallers (N_2_ = 114) based on prior history of falls. Six machine learning models (logistic regression, naïve bayes, decision tree, random forest, boosted tree and support vector machine) were proposed for faller classification. Results indicated that compared with non-fallers, fallers performed significantly worse on the test battery. In addition, the application of sensor data and support vector machine for faller classification achieved an overall accuracy of 89.4% with 92.7% sensitivity and 84.9% specificity. These findings suggest that wearable inertial sensor based systems show promise for elderly fall risk assessment, which could be implemented in clinical practice to identify “at-risk” individuals reliably to promote proactive fall prevention.

## Introduction

Falls present a substantial health problem among the older population. Approximately one-third of community-dwelling people aged 65 years or above experience at least one fall per year^1,2^. Subsequently, the frequency increases to nearly 50% for those individuals aged over 85 years^3,4^. 20~30% of falls result in injury or the need for medical attention, and consequently falls are the leading cause of death and non-fatal injury in older people^5^. Falls not only have serious negative physical consequences for older people regarding morbidity, mortality, and loss of independence but also result in ‘post-fall syndrome’ such as fear of falling, social isolation or depression^6^. The annual costs of falls in older populations and their consequences have been reported to range between 0.85% and 1.5% of the total healthcare expenses^7^, and fall-related injuries are considered as the “Global Burden of Disease” by the WHO^8^. Therefore, it is critical to prevent falls from happening.

Fall risk assessment is a useful prevention tool that identifies elderly individuals with high fall risks, and it can help for diagnosis and selection of appropriate interventions to ultimately reduce the occurrence of falls^9,10^. During the past two decades, a lot of research has been conducted to develop various fall risk assessment tools to screen out persons at a high risk of falls and construct different fall assessment models to estimate the probability of an outcome of fall. In general, the assessment tools have been evolved from questionnaires and simple clinical scales in clinical settings to sophisticated equipment in laboratory settings. Now, wearable sensor-based systems for fall risk assessment are an emerging trend. Even though questionnaires and clinical scales are often used for clinical fall risk assessments, most of them are subjective, qualitative and oversimplified to assess geriatric fall risk. Sophisticated equipment such as optical motion capture systems, force plates, and computerized dynamic posturography provide objective, quantitative measures for fall risk assessment. However, they are expensive, located in biomechanics or gait laboratory and require trained staff. Furthermore, previous studies also indicate that falls in older adults occur mostly in dynamic settings rather than static settings^11^ suggesting a need to investigate dynamic characteristics during daily living activities among older adults.

With technological advances in micro-electromechanical systems and integrated wireless sensing and communication, wearable sensors are growing in popularity because of their potential advantages over the expensive, cumbersome equipment in the research laboratory and subjective clinical scales. They could provide a quantifiable, objective indication of fall risk in the elderly population outside of the research laboratory. The wearable sensors include accelerometers, gyroscopes, magnetometers, interface pressure sensors, goniometers, etc. and they are typically low in cost and small in size that make them portable. Due to these properties, they have been utilized to measure the activities of daily life and even monitor the health status of the older people^12,13^.

A literature review^14^ evaluating the use of wearable inertial sensors to assess balance and risk of falls revealed that accelerometers and gyroscopes are the most frequently used sensors. Based on raw data recorded from these two types of sensors, more than 130 parameters (position and angle, angular velocity, linear acceleration, spatial and temporal variables, energy variables, etc.) have been reported in previous studies to distinguish fallers from non-fallers in older adult populations. Even though the accuracy of a few inertial sensors-based fall risk assessment tools can be up to 90% using clinical assessment tools as a reference, it is lower than 80% if using the golden standard of fall data as a reference^14^. In order to increase the accuracy of fall risk assessment tools, it is important to select the proper predictor variables and use appropriate methods to construct risk assessment models. However, for easy and quick administration, most of the studies selected the variables from one or two simple test tasks such as quiet standing, normal walking or the timed up and go test. As the underlying cause of a fall is complex and often multifactorial in nature, the variables from one or two tasks can only reflect a partial picture of fall, which oversimplifies geriatric fall risk and limits the accuracy of the fall assessment model. Thus, more work needs to be done to understand how wearable inertial sensor systems may be useful for assessing fall risk, which includes: (1) How to design and administer an appropriate test battery with wearable inertial sensors? (2) How to derive meaningful measures from sensor data and select optimal predictor variables for fall risk assessment? (3) How to develop scientific models to assess the fall risk accurately? The answers to those questions would pave the way for further development of wearable inertial sensor based fall risk assessment systems for practical applications.

Considering the challenge of population ageing and the substantial health problem among the elderly population from falls, the purpose of this study was to verify whether it is possible to distinguish accurately between older fallers and non-fallers, based on data from wearable inertial sensors collected during a specially designed test battery. The results from this work may be used to develop a wearable inertial sensor based multifactorial fall risk assessment system for identifying older individuals at risk of falling reliably for proactive fall prevention, thus help to reduce the fall risk for not only improving the quality of life of the general elderly population, but also reducing costs in healthcare systems.

## Methods

### Inertial sensors based experimental setup and a new test battery

**Figure 1:**
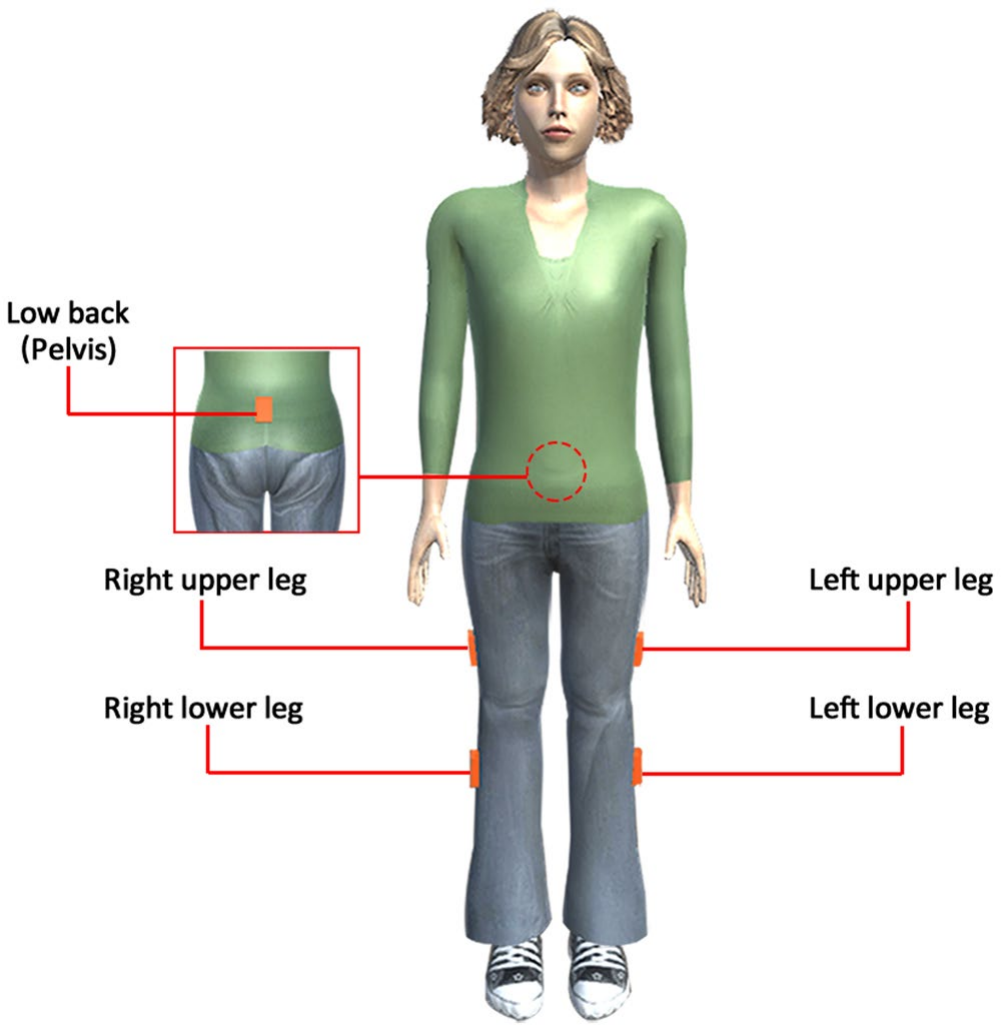
Inertial Sensor Configuration for Human Participants.

Five lightweight, miniature inertial sensors (weight: 10 g, size: 36 × 24.5 × 10 mm) from Xsens^15^ were placed over each participant’s low back, upper legs, and lower legs for sensor data collection (Fig. 1). Each inertial sensor had 9 degrees of freedom (3-axis acceleration, 3-axis angular velocity, and 3-axis magnetism) and its data sampling rate was 100 Hz.

In order to cover a range of major fall risk factors, a comprehensive but practical test battery for multifactorial fall risk assessment was custom designed based on a theoretical framework of the human balance system (physiological, psychological, and integrated functions). It is simple and quick for older people to perform with wearable inertial sensors or digital Apps. The whole test battery includes seven subtests (Table 1) and each subtest meets the following criteria: (1) Simple and quick to administer; (2) Feasible for older people to undertake; (3) Valid and reliable tests for assessing corresponding risk factors; (4) Quantitative measures, which should be mainly obtained from wearable inertial sensors of accelerometers and gyroscopes or digital Apps. These 7 subtests have been reported and tested in earlier studies: 1) Sensory integration test (SIT): Quiet standing at four different experimental conditions (Eyes open or close × firm or foam surface). Equilibrium score, calculated from anteroposterior and mediolateral tilt angle of low-back (approximation of center of mass) at different conditions, provides an indication of individual performance of the three sensory systems responsible for maintaining balance^16^. (2) Limits of stability (LOS)-forward reach. (3) Sit-to-stand five times (STS5). (4) Timed up and go test (TUG). (5) Motor function (MF)^17^. 6) Choice reaction test (CRT)^18^. And (7) Fear of falling test through computerized falls efficacy scale (FES)^19^. Performance on the first five subtests were measured using wearable inertial sensors and the last two subtests were through our developed digital Apps running on a Tablet PC.

**Table 1:** Representative Outcome Measures from 7 Subtests.

Subtests in The Test Battery	Representative Measures
Sensory Integration Test (SIT)	Time domain: Equilibrium score; RMS acceleration & angular velocity; jerk in acceleration or angular velocity. Frequency domain: median, centroid, and power spectral density of acceleration and angular velocity (anteroposterior, mediolateral directions)
Limits of Stability (LOS)	Forward reach distance; RMS angular velocity; Jerk in angular velocity
Sit-to-Stand Five Times (STS5)	Sit-stand-sit, sit-stand, and stand-sit transitions: durations; angular velocity; jerk
Timed Up and Go (TUG)	Gait pattern: gait velocity; step time & length; acceleration; angular velocity (anteroposterior, mediolateral and vertical directions). Turning phase: tuning time; angular velocity
Motor Function (MF)	Range of motion: knee flexion; knee extension
Choice Reaction Test (CRT)	Information processing speed; Simple reaction time
Computerized Falls Efficacy Scale (FES)	Falls efficacy scale international score

### Study population

All participants were volunteers recruited from social welfare centers in the city of Ulsan, South Korea. The eligibility criteria were as follows: female, age ≥65 years and living independently in the community. Only females were recruited in this study to avoid the influence of gender differences on fall risk, as females are more prone to fall and less active as compared to males^20^. In total, 196 community-dwelling older Korean women participated in this study (Table 2). Each participant gave informed consent prior to participation. All participants performed the seven subtests sequentially and completed the test battery within 30 minutes. The study was ethically approved by Ulsan National Institute of Science and Technology Institutional Review Board (No. 14–32-A). All these tests and measurements were carried out in accordance with relevant guidelines and regulations.

**Table 2:** Demographic Characteristics of Study Population.

Characteristics	Non-faller (N = 114) (mean ± SD)	Faller (N = 82) (mean ± SD)	Two-sample comparison (P value)
Age (years)	72.02 ± 4.17	72.35 ± 4.74	0.608
Height (cm)	154.83 ± 5.01	154.41 ± 5.31	0.577
Weight (kg)	58.01 ± 6.93	61.01 ± 8.05	0.006
BMI (kg/m^2^)	24.21 ± 2.74	25.56 ± 2.93	0.001

A fall was defined as ‘an unexpected loss of balance resulting in coming to rest on the floor, the ground, or an object below the knee level’^10,21^. Based on a self-reported history of falling in the past 5 years, older people were categorized as ‘fallers’ if they had experienced multiple falls or one injurious fall which required medical attention within one year prior to assessment^22^. Older participants who did not fulfill these criteria were considered as ‘non-fallers’. Based on the above criteria, there are 82 older fallers and 114 older non-fallers. Among all 82 fallers, 81.7% (67 fallers) of them reported no injury or only mild injuries such as a sprain, swelling and bruise. Even though 18.3% of fallers (15 fallers) reported moderate to severe injuries including fractures and major head trauma, they had largely recovered and were able to walk unaided prior to the test.

### Sensor data processing

The sensor data collected from all subtests were first filtered using a fourth order Butterworth low-pass filter with a cutoff frequency of 20 Hz^23^, which allows the signal to attenuate above the cut-off frequency to remove the noise in the time series data. Then various algorithms were developed for extracting meaningful fall risk measures from the sensor data. During this process, different sensors were used for different subtests due to their high relevance with the corresponding subtests and the specific movement patterns recorded during each subtest. For example, the data from the sensor positioned at the low-back was used for SIT and LOS tests; for STS5, the data from a sensor at the upper leg was used; for MF, the data from two sensors at both lower and upper legs were used; while for TUG, the data from three sensors at low-back, left and right lower legs were used.

**Figure 2:**
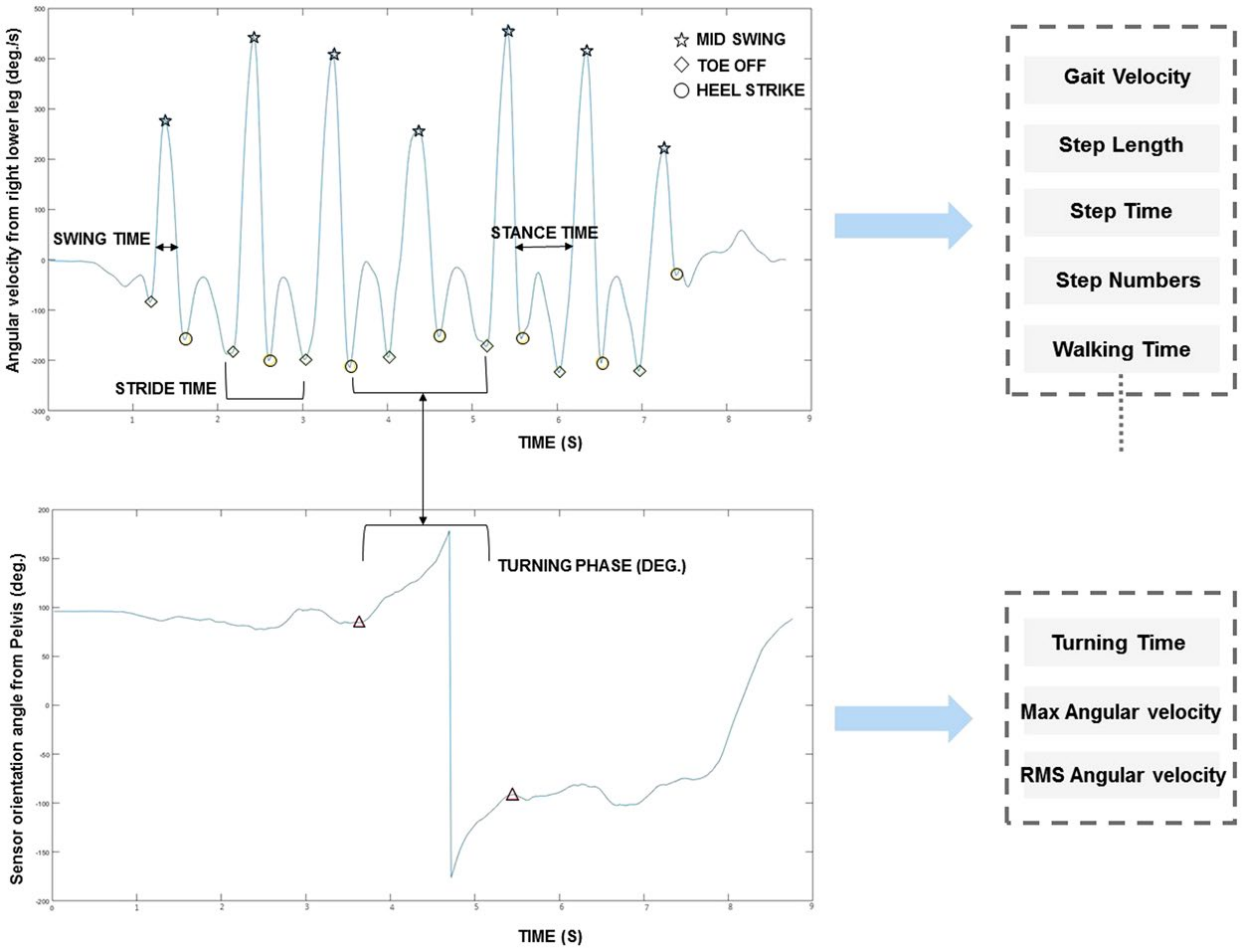
Algorithm Development for Timed Up and Go Test: From Feature Detection to Meaningful Measures Derivation.

To simplify the explanation, one of the most widely used tests-TUG is illustrated as a representative example and measures obtained from this test are based on raw sensor data and gait pattern, their definitions are mentioned elsewhere^24^. From the TUG test, the measures can be categorized as walk related measures (gait velocity, step length and walk time, etc.) and turn related measures (turning time and angular velocity)^13,25^. During walking, the angular velocity and acceleration from the low-back sensor were utilized for the calculation of raw sensor data-based measures. Similarly, angular velocity from the sensors at the left and right lower legs were used for the calculation of gait pattern measures. To derive the walk related measures from inertial sensors in the TUG test, the angular velocity from the sensor on the lower leg was used for identifying important features such as mid-swing, heel-strike and toe-off during walking (Fig. 2). First, the high peaks in each gait cycle will be identified as the mid-swings due to the largest angular velocity associated with the mid-swing in one cycle. Based on the gait cycle and biomechanical analysis, the troughs at the left and right side of the mid-swing should be considered as toe-off and heel-strike respectively^26^. Two consecutive toe-off or heel-strike points of the same foot determine one-step and time duration between them will be step time. Total distance divided by the number of steps is average step length. The time between the first toe-off and the last heel-strike excluding the turning time is walking time, which is further used for the gait velocity calculation. For the turn related measures in TUG, the sensor data from low-back such as angular velocity for measure calculation and orientation data for clear turning visualization was utilized (Fig. 2). The algorithm for the detection of the start of turning and the end of turning were similar as the detection of the mid-swing points. The start turning point will be when the mid-swing point detection criteria do not fulfill on both legs sensor data and end turning point when the mid-swing criteria do fulfill, but the peak value less than the other leg will be considered as end turning point. The duration between these two points is the turning time. The data from the low-back sensor within start turning and end turning points were used for the calculation of the angular velocity measures. Similarly, the algorithms were developed for other subtests to derive meaningful fall risk measures. In total, 155 measures (Supplementary Table S1) were obtained and some representative measures are listed in Table 1.

### Statistical analysis and fall risk modeling

First, two-sample t-tests were performed on 155 outcome measures from seven subtests to find the significant measures, which show statistical differences between faller and non-faller groups. Then a Receiver Operating Characteristic (ROC) analysis^18^ was carried out to examine the discriminative power of each significant measure on classifying fallers and non-fallers. In total, 38 measures were identified as statistically significant in both t-test and ROC analysis (Supplementary Table S2).

Supervised machine learning models incorporating the same set of 38 significant measures (Supplementary Table S2) as predictor variables and fall status as response variable were built to classify fallers and non-fallers. Typical machine learning models for automatic learning to make accurate classification based on experimental observations, included logistic regression, Naïve Bayes classifier, basic decision tree, boosted tree, random forest, and support vector machine. All of which were utilized to classify fallers and the classification accuracies of these models were then analyzed to identify their appropriateness for distinguishing fallers from non-fallers. Prediction accuracy of the models was assessed based on 10-fold cross-validation to avoid the problem of overfitting and to enhance the generalisability of the models^27^.

Raw sensor data were processed in Matlab (Mathworks, Natick, VA, USA) to calculate the derived measures. All statistical analyses were conducted using SPSS (v24, IBM Corp, Armonk, NY) and statistical significance was accepted at p < 0.05 and marginal significance was accepted at 0.05 ≤ p < 0.10. Classification models were constructed and validated in Weka 3.8^28^.

## Results

### Demographic characteristics of faller and non-faller groups

Table 2 shows the demographic characteristics of non-fallers and fallers. Even though there were no significant differences between the groups in terms of age (p = 0.608) and height (p = 0.577), fallers were significantly heavier (p = 0.006) and had higher BMI (p = 0.001) compared with non-fallers. According to the BMI classification from Centers for Disease Control and Prevention (underweight, BMI < 18.5 kg/m^2^; normal, 18.5 ≤ BMI < 25; overweight, 25 ≤ BMI < 30; and obese, BMI ≥30 kg/m^2^), 55% of fallers were categorized as overweight or obese and the rest were normal-weight. Only 40% of non-fallers were overweight or obese, and the remainder of the group were normal-weight. This result indicated that the faller group had higher prevalence of overweight and obesity than the non-faller group. This result agreed with the findings from previous studies^29–31^ which reported overweight/obesity as an important risk factor for elderly falls as it is not only negatively associated with physical activity levels, but also negatively impacts balance and postural control, especially at dynamic conditions such as ambulatory stumbling.

### Significant differences between faller and non-faller groups in the test battery

In the SIT test, when compared with non-fallers, fallers had a significantly lower equilibrium score (87.7 ± 5.75 vs. 89.9 ± 4.86, p = 0.002) for the visual system in mediolateral direction, higher root mean square (RMS) of angular velocity (1.47 ± 0.29 vs. 1.38 ± 0.22, p = 0.033) in anteroposterior, and jerk of acceleration in both anteroposterior (2.04 ± 0.61 vs. 1.78 ± 0.49, p = 0.001) and mediolateral directions (2.01 ± 0.61 vs. 1.81 ± 0.51, p = 0.016). For the vestibular system, jerk of acceleration in anteroposterior direction was higher for fallers with a marginal significance (3.23 ± 2.04 vs. 2.78 ± 1.24, p = 0.063). For standing on a foam surface with eyes open, fallers had a significantly lower equilibrium score in the mediolateral direction (82.03 ± 5.20 vs. 84.04 ± 4.34, p = 0.005) and higher RMS of acceleration in both the anteroposterior (0.02 ± 0.01 vs. 0.01 ± 0.01, p = 0.033) and mediolateral (0.007 ± 0.002 vs. 0.006 ± 0.001, p = 0.002) directions. While standing on the firm surface, fallers showed significantly lower jerk of acceleration in the anteroposterior direction in both the eyes open (0.039 ± 0.009 vs. 0.043 ± 0.012, p = 0.009) and eyes closed conditions (0.044 ± 0.012 vs. 0.049 ± 0.016, p = 0.028). In the LOS test during forward reach, fallers had significantly lower jerk of angular velocity in the mediolateral direction (24.96 ± 2.79 vs. 26.24 ± 3.15, p = 0.004). In the STS5 test, fallers took significantly longer to complete the sit-stand-sit (2.44 ± 0.78 vs. 2.05 ± 0.43, p < 0.001), stand-sit (1.32 ± 0.55 vs. 1.08 ± 0.26, p < 0.001), and sit-stand transitions (1.17 ± 0.39 vs. 0.97 ± 0.19, p < 0.001) as compared to non-fallers. In all three transitions, fallers had significantly lower angular velocity (p = 0.001, 0.003 and < 0.001 for sit-stand-sit, stand-sit and sit-stand respectively) and jerk of angular velocity (p = 0.004, 0.025 and 0.001 for sit-stand-sit, stand-sit and sit-stand respectively).

In the TUG test, fallers had a significantly shorter step length (0.36 ± 0.04 vs. 0.39 ± 0.04, p < 0.001), longer step time (1.08 ± 0.13 vs. 1.04 ± 0.08, p = 0.042), and slower gait velocity (0.68 ± 0.12 vs. 0.77 ± 0.11, p < 0.001) during the walking phase. Fallers also showed significantly lower RMS of angular velocity in the anteroposterior (p = 0.005), mediolateral (p = 0.001) and vertical (p < 0.001) directions. During the turning phase, fallers spent significantly more time completing the turn (2.43 ± 0.61 vs. 2.11 ± 0.43, p < 0.001), had a lower angular velocity in terms of RMS (76.63 ± 13.83 vs. 84.64 ± 10.33, p < 0.001) and maximum (104.93 ± 16.31 vs. 113.78 ± 10.89, p < 0.001) than the non-fallers. In the MF test, the ranges of motion in the knee joint during flexion (124.95 ± 12.99 vs. 131.78 ± 10.89, p < 0.001) and extension (3.69 ± 1.82 vs. 4.29 ± 1.55, p = 0.013) were significantly smaller for fallers. In the CRT test, the information processing speed was significantly lower in fallers (5.82 ± 1.27 vs. 7.09 ± 1.28, p < 0.001) compared with non-fallers even though there was no significant difference on simple reaction time (p = 0.169). In terms of fear of falling, fallers had significantly higher FES scores compared with non-fallers (14.96 ± 4.92 vs. 9.73 ± 2.98, p < 0.001), indicating a greater concern about falling. Table 3 shows the top ten significant measures from the 7 subtests used to differentiate fallers from non-fallers. The rank of significant measures is based on the mean decrease accuracy in the random forest^32^, with consideration of area under the ROC curve (AUC) and p-value from statistical t-tests. Detailed results of all 38 significant measures are provided in Supplementary Table S2.

**Table 3:** Top 10 Important Measures From 7 Subtests for Faller Classification.

Measures	Non-fallers, N = 114 (mean ± SD)	Faller, N = 82 (mean ± SD)	t-tests (P value)	Area under ROC curve (AUC)	Mean Decrease Accuracy
Fall Efficacy Scale (FES) Score	9.73 ± 2.98	14.96 ± 4.92	<0.001	0.834	38.57
Information Processing Speed (bit/sec.)	7.09 ± 1.28	5.82 ± 1.27	<0.001	0.753	28.52
Step Length (m)	0.39 ± 0.04	0.36 ± 0.04	<0.001	0.703	19.33
Gait Velocity (m/sec.)	0.77 ± 0.11	0.68 ± 0.12	<0.001	0.693	14.72
Stand-Sit Jerk (rad/sec.³)	1507.23 ± 519.93	1318.21 ± 650.68	0.025	0.625	11.69
Knee Extension Range (deg.)	4.29 ± 1.55	3.69 ± 1.82	0.013	0.624	11.65
Sit-Stand-Sit Jerk (rad/sec.³)	1592.92 ± 553.47	1341.82 ± 651.17	0.004	0.646	11.44
Turning Angular Velocity MAX (rad/sec.)	113.78 ± 10.89	104.93 ± 16.31	<0.001	0.659	11.17
Visual-Equilibrium Score Mediolateral	89.1 ± 4.3	87.0 ± 5.2	0.002	0.623	10.56
Knee Flexion Range (deg.)	131.73 ± 13.38	124.95 ± 12.99	<0.001	0.660	10.19

Overall, fallers showed worse performances of visual and vestibular systems, a smaller range of motion at the knee joint, a lower information processing speed, a higher fear of falling, and experienced more difficulties when completing the relatively complex tasks such as TUG, LOS, and STS5.

### Accuracy of fall classification models

Table 4 summarizes the classification performances of six fall classification models. Logistic regression (LR) and Naïve Bayes (NB) classifiers presented almost identical performances on fall classification, with overall accuracy around 80%, 87% sensitivity and 69% specificity. Similarly, the basic decision tree (DT) had ~82% overall accuracy with 87% sensitivity and 73% specificity. The support vector machine (SVM), boosted tree (BT), and random forest (RF) showed better classification performances: overall accuracy was higher than 86% with at least 90% sensitivity and 78% specificity. The SVM gave the best classification performance of 89.4% overall accuracy with 92.7% sensitivity and 84.9% specificity. In addition, the sensitivities of all classification models were higher than 87%, indicating that the pre-trained machine learning models are sensitive when identifying older people who are at high risk of falling.

**Table 4:** Overall Accuracy, Sensitivity, and Specificity of Six Classification Models Based On 10-Fold Cross-Validation.

Classification Models	Overall Accuracy, % (mean ± SD)	Sensitivity, % (mean ± SD)	Specificity, % (mean ± SD)
Support Vector Machine	89.42 ± 4.82	92.67 ± 6.17	84.90 ± 8.68
Boosted Tree	87.09 ± 5.56	91.23 ± 6.71	81.37 ± 9.37
Random Forest	86.39 ± 5.41	92.23 ± 5.49	78.06 ± 10.63
Decision Tree	81.64 ± 6.09	87.25 ± 7.56	73.29 ± 10.62
Naïve Bayes	80.05 ± 6.11	87.91 ± 6.60	69.16 ± 11.80
Logistic Regression	79.70 ± 6.37	87.24 ± 6.75	69.23 ± 11.94

Further two-sample t-tests on overall classification accuracy of machine learning models between SVM and 5 other models (BT, RF, DT, NB, and LR) showed that SVM achieved significantly higher accuracy than all of the other models. SVM had higher accuracy of 2.3% (95% CI: 0.9% to 3.8%, p = 0.002) than BT, 3.0% (95% CI: 1.6% to 4.5%, p < 0.001) than RF, 7.8% (95% CI: 6.3% to 9.3%, p < 0.001) than DT, 9.4% (95% CI: 7.8% to 10.9%, p < 0.001) than NB, and 9.7% (95% CI: 8.1% to 11.3%, p < 0.001) than LR.

## Discussion

In order to assess the major intrinsic fall risk factors systematically with the inertial sensors and digital Apps, a comprehensive test battery consisting of 7 subtests was proposed. Three subtests were designed for evaluating physiological risk factors: sensory integration test for assessing risk factors of sensory inputs from human visual, vestibular and somatosensory subsystems; choice reaction test for assessing risk factors of central processing system; and motor function test for motor system related risk factors. For psychological risk factors, a computerized FES questionnaire was proposed to assess the fear of falling. An additional three subtests were designed to assess the integrated functions: Limits of stability and sit to stand five times tests were utilized for the postural stability, adjustment and response assessment; Timed up and go test was utilized for the gait mobility assessment. Experimental results on 196 community-dwelling older women showed that many major fall risk factors were reflected within the inertial sensor data obtained during the specially designed test battery. During quiet standing in the sensory integration test, the faller group had a significantly lower equilibrium score and greater magnitude of body sway (higher RMS of angular velocity, jerk of acceleration) than the non-faller group for both visual and vestibular systems. With poor input from the visual senses, the ability to control balance and avoid obstacles became impaired due to misinterpretation of spatial information and weakened depth perception. Due to attrition of neural and sensory hair cells, vestibular dysfunction is common in older people^33^. Interestingly, there were no significant differences in the somatosensory measures between fallers and non-fallers, which may be because all the participants in this study were in relatively good health and had no somatosensory loss from peripheral neurology^34^. In the choice reaction test, the fallers had significantly lower information processing speed than non-fallers, even though the difference on the simple reaction time was not significant. These findings suggest low information processing speed or cognitive impairment is an important risk factor for falling in older people. This is reasonable since slow reactions of central processing system delay the sensory integration process and the corrective responses of muscles and joints during the critical situations such as slips, trips and missteps, and result in falls^18,35^.

In the motor function test, fallers had a significantly smaller range of motion (ROM) at the knee joint, indicating less flexibility in the knee joints, which was associated with an increased fall risk^36^. Adequate flexibility and muscle function of the ankle, knee and hip joints are essential for human mobility and balance since they are key joints in postural control strategies for fall prevention^37,38^. Duncan *et al*.^39^ found a significant decrease of ROM of knee flexion in the high mobility group when compared with the low mobility group for the elderly men without significant diseases. Zamanian^40^ investigated the effectiveness of a water exercise in 30 elderly women with knee osteoarthritis and reported that after a 12 weeks of water exercise the ROM of knee was significantly improved and the fall risk was also significantly reduced. These findings suggest a link between decreased ROM of the knee and falls in the elderly and interventions addressing ROM deficits can decrease the risk of falls^41^. In psychological aspects, computerized FES score was significantly higher in fallers, indicating higher fear of falling. These results are consistent with previous studies which reported that the major risk factors for developing a fear of falling are experiencing a previous fall, being female and being older^42^. In addition, 50%-60% of reported fallers experienced fear of falling in several community-dwelling samples^43^. Fear of falling is one of the key symptoms of ‘post-fall syndrome’ in the elderly population, resulting in a decline in physical and cognitive performance, an increased risk of falling and poor quality of life^44^.

In LOS, STS5 and TUG subtests for integrated functions, fallers experienced more difficulty to perform the designated tasks. This was reflected from significantly longer task completion time, lower acceleration, angular velocity, and jerk. The higher acceleration, angular velocity or jerk indicated that the participants successfully performed the relatively complex tasks quickly and thus had better balance ability. For example, in the TUG test, a lower gait velocity, longer step time, and shorter step length were observed in fallers during the walking phase. During the turning phase, fallers took significantly longer turning time with smaller turning angular velocity than non-fallers. These were largely consistent with the results obtained in many earlier studies^25^.

Six typical supervised machine-learning models were developed to classify older fallers and non-fallers based on the same set of 38 significant measures from inertial sensor data and digital Apps. The overall classification accuracy of the models varied from 79.7% to 89.4% (Table 4), where linear and simple models such as logistic regression, naïve Bayes and basic decision tree had relatively lower classification accuracies (~80%) and advanced models including boosted tree, random forest and support vector machine showed excellent accuracies (>86%) on classifying older fallers and non-fallers. Different performances on fall classification would most likely be caused by different flexibilities of classification models. Linear models have a more restrictive assumption and a basic tree model has a relatively lower flexibility when compared with those advanced tree models (boosted tree, random forest) and support vector machine. Advanced models with good flexibility have the advantage of being able to handle high-dimensional data with correlated variables^32,45^ and good flexibility of a model is associated with high accuracy^27^.

The overall accuracy of fall classification models in this study was higher than or at least comparable to those of previous studies (Table 5)^5,22,46–53^, which also used inertial sensors to classify older fallers and non-fallers and evaluated the classification accuracy based on the fall data. Most of earlier fall classification models had overall accuracies lower than 80%. The improved model accuracy from this study could be explained by the fact that we designed a comprehensive test battery to cover major fall risk factors and included significant measures from different aspects (physiological, psychological and integrated functions; Supplementary Table S2) into the classification models. However, most of previous studies only included the measures from one single physical test, such as quiet standing^46^, walking short distances^47,48^, STS5^22^, TUG^49^, or a combination of two or three of the aforementioned tests^5,50^. Adding significant measures associated with different major fall risk factors such as cognitive impairment and psychological aspects such as a fear of falling significantly improved the model accuracy, especially for those advanced fall classification models with high flexibility, as different predictive fall risk variables can contribute to final classification in different models. Of course, a larger number of inertial sensors (5) used in this study to obtain more informative data could be another potential cause. In many previous studies, only one inertial sensor placed close to the center of mass (the low-back, pelvis or L3-L5) can give overall body sway measures during quiet standing and has high acceptance for prolonged use^54^, however it is difficult to provide detailed information about human movement patterns when performing relatively complex tests such as TUG and STS5, therefore in our study five inertial sensors were placed at each lower body segment to record the minor changes at the low-back, upper and lower legs. Elderly fall risk classification is a complex problem where differences between “at risk” and “low risk” individuals on some aspects could be subtle and varied in many instances^55^.

**Table 5:** Previous Studies on Elderly Fall Risk Assessment with Wearable Inertial Sensors.

Studies	Sensors & Locations	Experimental Participants	Testing Tasks & Measures	Classification Models	References for Fall Classification	Validation Method	Overall Accuracy in % (Sensitivity & Specificity)
Howcroft, *et al*.^48^	4 tri-axial accelerometers (X16-1C): left and right shank, head, and pelvis; Pressure sensing insole (F-scan)	100 (56 females and 44 males): age 75.5 ± 6.7	7.62 m walk: temporal, center of pressure & frequency-based measures	Support vector machine, naïve Bayesian, multi-layer neural network	Fall history	75:25 single stratified holdout & repeated random sampling	70–78 (Sens: 16–55, Spec: 68–91)
Greene, *et al*.^52^	2 inertial sensors (Accelerometer and gyroscope): left and right anterior shanks	422 (308 females and 114 males); age 73.6 ± 7.4	(1) Timed up and go; (2) Clinical based measures	Logistic regression	Fall history	Leave-one-out- cross validation, Ten-fold cross validation	59–76 (Sens: 36–74, Spec: 62–86)
Similä, *et al*.^53^	2 accelerometers (GCDC X16-2): lower back (L3-L5) & front right hip	35 females; age 73.9 ± 5.4	(1) Berg Balance Scale; (2) Timed up and go: walk time, step time, step frequency, etc.; (3) 4 m walk	Generalized linear models	Prospective falls	Ten-fold cross validation	69–79 (Sens: 80, Spec: 67–73)
Doheny, *et al*.^22^	2 Shimmer tri-axial accelerometers: lateral right thigh and sternum	39 (11 females and 28 males); age 73.6 ± 6.6	Sit to stand five times: RMS acceleration, jerk, etc.	Logistic regression	Fall history	Leave-one-out- cross validation	74.4 (Sens: 69, Spec: 80)
Bautmans, *et al*.^47^	1 Accelerometer (DynaPort MiniMod): pelvis	81 elderly subjects; age 79.9 ± 5.2	(1) 18 m walk: step time asymmetry; (2) Muscle force: grip strength & endurance of the dominant hand	Logistic regression & ROC curve	Fall history	Not specified	77 (Sens: 78, Spec: 78)
Greene, *et al*.^50^	5 Shimmer sensors: one on each shin, right thigh, lower back, and sternum	124 (91 females and 33 males): age 75.9 ± 6.6	(1)Timed up and go; (2) Sit to stand 5 times; (3) Quiet standing	Support vector machine	Fall history	Mean cross-validated	83 (Sens: 79, Spec: 83)
Marschollek, *et al*.^51^	1 Freescale RD3152MMA7260Q 3-axis accelerometer: waist	110 patients (81 females and 29 males): age 80	(1) Timed up and go: pelvic sway, step length, No. of steps; (2) STRATIFY score; (3) Barthel index.	Decision tree	Fall history	Ten-fold cross-validation	83–90 (Sens: 39–58, Spec: 98–100)
Greene, *et al*.^46^	1 Shimmer sensor at L3 vertebra, 1 Tactex S4 HD pressure mat	120 (63 females and 57 males): age 73.7 ± 5.8	Quiet standing: RMS acceleration, angular velocity, median frequency, etc.	Support vector machine	Fall history	Ten-fold cross validation	71.5 (Sens: 65, Spec: 68)
Marschollek, *et al*.^49^	1 Freescale RD3152MMA7260Q 3-axis accelerometer: waist	50 patients (37 females and 13 males): age 81.3	(1)Timed up and go: kinetic energy, pelvic sway, step length, etc. (2) STRATIFY score	Logistic regression	Prospective falls	Ten-fold cross-validation	70–72 (Sens: 58, Spec: 78)
Marschollek, *et al*.^5^	1 Freescale RD3152MMA7260Q 3-axis accelerometer: waist	50 patients (37 females and 13 males): age 81.3	(1) Timed up and go: kinetic energy, pelvic sway, step length, etc.; (2) 20 m walk; (3) STRATIFY score; (4) Barthel index	Logistic regression, decision tree	Prospective falls	Ten-fold cross-validation	65–80 (Sens: 58–74, Spec: 82–96)

It is worthwhile to mention that a good balance between the sensitivity and specificity is very important when classifying fallers. In general, the higher the sensitivity, the lower the specificity, and vice versa^56^. However, a fall risk assessment tool with a high sensitivity but too low specificity will result in many older people who are not at risk of falls being classified as having a high risk of falls (false positive) and are then subject to further investigation. On the other hand, a fall risk assessment tool with a high specificity but too low sensitivity will result in many older people who actually are at high fall risk being classified as having a low risk of falls (false negative) and then miss the chance to undertake an in-depth examination^56^. Most earlier studies (Table 5) reported higher specificity than sensitivity^22,48,54^ and the sensitivity was generally lower than 70%, which resulted in more than 30% older people at high risk of falling being wrongly classified as non-fallers. In this study, the developed fall classification model based on SVM can achieve sensitivity of 92.7% and specificity of 84.9%, which showed not only excellent overall performance, but also good balance between sensitivity and specificity.

This study has some limitations. First, our proposed test battery only focused on the major intrinsic factors for fall risk assessment, without consideration of the influence of extrinsic factors on the occurrence of the fall. Second, retrospective falls were used in this study for classifying the older fallers and developing fall classification models. Even though a history of falls was reported to have the strongest association with increased fall risk^57,58^ and older adults who fall once were two to three times as likely to fall again within a year^59^, prospective studies are needed to confirm the predictive ability of the developed model for future falls in the older people. Third, the expansion of the proposed method and developed fall classification models in this study to an easy-to-use tool (e.g., a fully wireless mobile platform such as a smartphone or tablet) for practical assessment of elderly fall risk, is necessary. Lastly, understanding how to extend the fall risk assessment to diagnose risk and to identify underlying risk factors or specific impairments that increase fall risk, should be further investigated to enhance the clinical value of wearable-inertial sensor based fall risk assessments.

## Conclusions

In this study, we proposed an innovative method for multifactorial fall risk assessment based on data from wearable inertial sensors, collected during a specially designed test battery. To verify the feasibility of the proposed method, 196 community-dwelling Korean older women were recruited for an experimental study. The results showed that data obtained from wearable inertial sensors distinguished accurately between retrospective fallers and non-fallers, with overall accuracy of 89.4% (92.7% sensitivity and 84.9% specificity) when the support vector machine was applied for classification. These findings indicated that wearable inertial sensor based systems show great promise for elderly fall risk assessment. The proposed method and the developed fall classification models may be implemented in clinical practice to identify “at-risk” individuals reliably so that appropriate interventions and prevention programs can be applied to reduce the risk of falling in older adults and ultimately improve their quality of life.

## Electronic supplementary material

Supplementary Tables

## Data Availability

The statistical data of all 155 outcome measures from the test battery and 38 significant measures between faller and non-faller groups that support the major findings of this study are summarized in the Supplementary Tables S1 and S2. The completed data are not publicly available due to Institute Review Board related matters, but are available from the corresponding author (Shuping Xiong shupingx@kaist.ac.kr) upon reasonable request.
