# Supplementary material for: Application of Wearable Inertial Sensors and A New Test Battery for Distinguishing Retrospective Fallers from Non-fallers among Community-dwelling Older People
Source: Sci Rep. 2018 Nov 5;8:16349. doi: 10.1038/s41598-018-34671-6 (PMC6218502; doi:10.1038/s41598-018-34671-6)
Supplement: Supplementary file 1 — Supplementary Tables [file 41598_2018_34671_MOESM1_ESM.docx]

**Application of Wearable Inertial Sensors and A New Test Battery** **for Distinguishing Retrospective Fallers from Non-fallers among Community-dwelling Older People**

Hai Qiu^1 §^, Rana Zia Ur Rehman^1 §^, Xiaoqun Yu^1^ and Shuping Xiong^1*^

^1^Human Factors and Ergonomics Laboratory, Department of Industrial & Systems Engineering, Korea Advanced Institute of Science and Technology (KAIST), Daejeon, South Korea

**Supplementary Table S1:** 155 Outcome Measures from Seven Subtests

| **Subtests** | **Outcome Measures** | **Non-fallers** (mean ± SD) | **Fallers**  (mean ± SD) | **T test (p)** | **AUC** |
| --- | --- | --- | --- | --- | --- |
| Sensory Integration Test (SIT) | EOFS – Acceleration AP RMS | 0.007 ± 0.002 | 0.007 ± 0.003 | 0.150 | 0.541 |
|  | EOFS – Acceleration ML RMS | 0.0023 ± 0.001 | 0.0025 ± 0.001 | 0.090 | 0.569 |
|  | EOFS – AngVel AP RMS | 0.527 ± 0.046 | 0.526 ± 0.043 | 0.671 | 0.511 |
|  | EOFS – AngVel ML RMS | 1.069 ± 0.079 | 1.081 ± 0.091 | 0.173 | 0.532 |
|  | EOFS – ES AP | 82.918 ± 4.421 | 82.237 ± 5.110 | 0.170 | 0.533 |
|  | EOFS – ES ML | 93.469 ± 1.845 | 93.338 ± 1.987 | 0.502 | 0.516 |
|  | EOFS – Jerk AP acc. | 0.043 ± 0.013 | 0.039 ± 0.009 | <0.001 | 0.589 |
|  | EOFS – Jerk ML acc. | 0.044 ± 0.014 | 0.041 ± 0.012 | 0.040 | 0.547 |
|  | EOFS – Jerk AP AngVel | 4.789 ± 0.638 | 4.641 ± 0.422 | 0.006 | 0.560 |
|  | EOFS – Jerk ML AngVel | 5.863 ± 1.235 | 5.592 ± 0.774 | 0.008 | 0.560 |
|  | EOFS – Med. Fre. acc. AP | 0.279 ± 0.096 | 0.271 ± 0.089 | 0.431 | 0.530 |
|  | EOFS – Med. Fre. acc. ML | 2.014 ± 1.642 | 1.398 ± 1.289 | <0.001 | 0.605 |
|  | EOFS – Med. Fre. AngVel AP | 3.135 ± 1.109 | 2.805 ± 1.058 | 0.003 | 0.588 |
|  | EOFS – Med. Fre. AngVel ML | 0.644 ± 0.404 | 0.582 ± 0.306 | 0.086 | 0.540 |
|  | EOFS – PSD acc AP | 0.003 ± 0.002 | 0.003 ± 0.002 | 0.057 | 0.536 |
|  | EOFS – PSD acc ML | 0.0004 ± 0.0003 | 0.0004 ± 0.0003 | 0.384 | 0.535 |
|  | EOFS – PSD AngVal AP | 0.043 ± 0.026 | 0.044 ± 0.022 | 0.733 | 0.526 |
|  | EOFS – PSD AngVal ML | 0.243 ± 0.140 | 0.267 ± 0.183 | 0.161 | 0.534 |
|  | EOFS – CF acc. AP | 4.521 ± 1.251 | 4.212 ± 1.248 | 0.017 | 0.571 |
|  | EOFS – CF acc ML | 5.483 ± 0.997 | 5.431 ± 0.997 | 0.616 | 0.519 |
|  | EOFS – CF AngVal AP | 6.263 ± 1.015 | 6.158 ± 1.035 | 0.319 | 0.529 |
|  | EOFS – CF AngVal ML | 4.838 ± 0.833 | 4.708 ± 0.920 | 0.150 | 0.550 |
|  | ECFS – Acceleration AP RMS | 0.008 ± 0.002 | 0.008 ± 0.003 | 0.022 | 0.560 |
|  | ECFS – Acceleration ML RMS | 0.003 ± 0.001 | 0.003 ± 0.001 | 0.236 | 0.540 |
|  | ECFS – AngVel AP RMS | 0.543 ± 0.049 | 0.537 ± 0.071 | 0.645 | 0.509 |
|  | ECFS – AngVel ML RMS | 1.129 ± 0.102 | 1.146 ± 0.113 | 0.116 | 0.557 |
|  | ECFS – ES AP | 80.274 ± 5.794 | 79.212 ± 6.248 | 0.088 | 0.546 |
|  | ECFS – ES ML | 92.849 ± 2.508 | 92.839 ± 2.654 | 0.968 | 0.508 |
|  | ECFS - Jerk AP acc | 0.049 ± 0.016 | 0.044 ± 0.012 | 0.028 | 0.574 |
|  | ECFS – Jerk ML acc. | 0.048 ± 0.019 | 0.044 ± 0.018 | 0.063 | 0.543 |
|  | ECFS – Jerk AP AngVel | 4.936 ± 0.866 | 4.803 ± 0.693 | 0.092 | 0.533 |
|  | ECFS – Jerk ML AngVel | 6.177 ± 1.749 | 5.848 ± 0.974 | 0.018 | 0.553 |
|  | ECFS – Med. Fre. acc. AP | 0.301 ± 0.109 | 0.289 ± 0.091 | 0.221 | 0.525 |
|  | ECFS – Med. Fre. acc. ML | 1.929 ± 1.663 | 1.374 ± 1.272 | <0.001 | 0.603 |
|  | ECFS – Med. Fre. AngVel AP | 3.066 ± 0.997 | 2.701 ± 1.036 | 0.001 | 0.593 |
|  | ECFS – Med. Fre. AngVel ML | 0.611 ± 0.358 | 0.546 ± 0.254 | 0.037 | 0.553 |
|  | ECFS – PSD acc AP | 0.004 ± 0.004 | 0.004 ± 0.003 | 0.509 | 0.544 |
|  | ECFS – PSD acc ML | 0.001 ± 0.001 | 0.001 ± 0.001 | 0.601 | 0.515 |
|  | ECFS – PSD AngVal AP | 0.047 ± 0.030 | 0.052 ± 0.060 | 0.256 | 0.536 |
|  | ECFS – PSD AngVal ML | 0.353 ± 0.316 | 0.363 ± 0.230 | 0.731 | 0.539 |
|  | ECFS – CF acc. AP | 4.501 ± 1.363 | 4.325 ± 1.318 | 0.203 | 0.530 |
|  | ECFS – CF acc ML | 5.425 ± 0.924 | 5.187 ± 0.894 | 0.011 | 0.572 |
|  | ECFS – CF AngVal AP | 6.152 ± 1.027 | 6.247 ± 1.082 | 0.374 | 0.529 |
|  | ECFS – CF AngVal ML | 4.701 ± 0.848 | 4.641 ± 0.964 | 0.520 | 0.533 |
|  | EOMS - Acceleration AP RMS | 0.01 ± 0.01 | 0.02 ± 0.01 | 0.033 | 0.583 |
|  | EOMS - Acceleration ML RMS | 0.006 ± 0.001 | 0.007 ± 0.002 | 0.002 | 0.618 |
|  | EOMS – AngVel AP RMS | 0.73 ± 0.13 | 0.77 ± 0.16 | 0.062 | 0.571 |
|  | EOMS – AngVel ML RMS | 1.282 ± 0.205 | 1.321 ± 0.231 | 0.081 | 0.570 |
|  | EOMS - ES AP | 72.38 ± 7.11 | 70.49 ± 6.96 | 0.066 | 0.591 |
|  | EOMS - ES ML | 84.04 ± 4.34 | 82.03 ± 5.20 | 0.005 | 0.615 |
|  | EOMS – Jerk AP acc. | 0.078 ± 0.040 | 0.081 ± 0.034 | 0.510 | 0.538 |
|  | EOMS – Jerk ML acc. | 0.079 ± 0.041 | 0.081 ± 0.034 | 0.599 | 0.535 |
|  | EOMS – Jerk AP AngVel | 6.654 ± 2.131 | 6.665 ± 1.880 | 0.959 | 0.514 |
|  | EOMS – Jerk ML AngVel | 8.018 ± 4.773 | 7.943 ± 2.607 | 0.854 | 0.533 |
|  | EOMS – Med. Fre. acc. AP | 0.361 ± 0.281 | 0.353 ± 0.119 | 0.753 | 0.534 |
|  | EOMS – Med. Fre. acc. ML | 0.525 ± 0.745 | 0.384 ± 0.185 | 0.006 | 0.583 |
|  | EOMS – Med. Fre. AngVel AP | 1.021 ± 0.695 | 0.868 ± 0.527 | 0.014 | 0.563 |
|  | EOMS – Med. Fre. AngVel ML | 0.779 ± 0.572 | 0.736 ± 0.520 | 0.442 | 0.528 |
|  | EOMS – PSD acc AP | 0.008 ± 0.004 | 0.009 ± 0.006 | 0.001 | 0.596 |
|  | EOMS – PSD acc ML | 0.004 ± 0.002 | 0.005 ± 0.004 | <0.001 | 0.605 |
|  | EOMS – PSD AngVal AP | 0.301 ± 0.231 | 0.396 ± 0.370 | 0.004 | 0.579 |
|  | EOMS – PSD AngVal ML | 0.719 ± 0.714 | 0.869 ± 0.853 | 0.059 | 0.587 |
|  | EOMS – CF acc. AP | 4.217 ± 1.030 | 4.430 ± 1.253 | 0.075 | 0.541 |
|  | EOMS – CF acc ML | 4.649 ± 0.995 | 4.390 ± 0.901 | 0.009 | 0.571 |
|  | EOMS – CF AngVal AP | 4.814 ± 0.911 | 4.319 ± 0.844 | 0.014 | 0.576 |
|  | EOMS – CF AngVal ML | 4.319 ± 0.919 | 4.197 ± 0.858 | 0.184 | 0.531 |
|  | ECMS – Acceleration AP RMS | 0.016 ± 0.008 | 0.016 ± 0.005 | 0.666 | 0.552 |
|  | ECMS – Acceleration ML RMS | 0.010 ± 0.004 | 0.010 ± 0.004 | 0.232 | 0.556 |
|  | ECMS – AngVel AP RMS | 0.933 ± 0.329 | 0.963 ± 0.339 | 0.365 | 0.532 |
|  | ECMS – AngVel ML RMS | 1.690 ± 1.327 | 1.703 ± 0.740 | 0.911 | 0.553 |
|  | ECMS – ES AP | 57.760 ± 18.255 | 58.392 ± 12.846 | 0.688 | 0.529 |
|  | ECMS – ES ML | 75.938 ± 11.407 | 75.320 ± 9.455 | 0.571 | 0.546 |
|  | ECMS – Jerk AP acc. | 0.123 ± 0.083 | 0.127 ± 0.088 | 0.657 | 0.530 |
|  | ECMS – Jerk ML acc. | 0.124 ± 0.104 | 0.127 ± 0.132 | 0.809 | 0.519 |
|  | ECMS – Jerk AP AngVel | 9.280 ± 5.755 | 9.402 ± 7.383 | 0.854 | 0.504 |
|  | ECMS – Jerk ML AngVel | 11.019 ± 9.212 | 10.761 ± 6.717 | 0.760 | 0.523 |
|  | ECMS – Med. Fre. acc. AP | 0.416 ± 0.266 | 0.418 ± 0.258 | 0.953 | 0.519 |
|  | ECMS – Med. Fre. acc. ML | 0.632 ± 0.856 | 0.478 ± 0.524 | 0.029 | 0.585 |
|  | ECMS – Med. Fre. AngVel AP | 1.116 ± 0.860 | 0.934 ± 0.607 | 0.014 | 0.555 |
|  | ECMS – Med. Fre. AngVel ML | 0.749 ± 0.579 | 0.736 ± 0.483 | 0.808 | 0.510 |
|  | ECMS – PSD acc AP | 0.018 ± 0.021 | 0.020 ± 0.039 | 0.453 | 0.542 |
|  | ECMS – PSD acc ML | 0.009 ± 0.010 | 0.010 ± 0.011 | 0.189 | 0.557 |
|  | ECMS – PSD AngVal AP | 0.693 ± 0.909 | 0.819 ± 1.302 | 0.262 | 0.545 |
|  | ECMS – PSD AngVal ML | 2.707 ± 15.338 | 2.650 ± 11.592 | 0.968 | 0.558 |
|  | ECMS – CF acc. AP | 4.208 ± 1.283 | 4.120 ± 1.134 | 0.483 | 0.515 |
|  | ECMS – CF acc ML | 4.601 ± 1.154 | 4.523 ± 0.930 | 0.476 | 0.512 |
|  | ECMS – CF AngVal AP | 4.652 ± 0.855 | 4.477 ± 0.849 | 0.045 | 0.565 |
|  | ECMS – CF AngVal ML | 4.131 ± 0.992 | 4.157 ± 0.844 | 0.793 | 0.525 |
|  | VIS – Acceleration AP RMS | 1.634 ± 0.572 | 1.681 ± 0.570 | 0.420 | 0.534 |
|  | VIS – Acceleration ML RMS | 3.149 ± 1.051 | 3.346 ± 1.191 | 0.084 | 0.550 |
|  | VIS – AngVel AP RMS | 1.38 ± 0.22 | 1.47 ± 0.29 | 0.033 | 0.581 |
|  | VIS – AngVel ML RMS | 1.199 ± 0.156 | 1.221 ± 0.167 | 0.184 | 0.558 |
|  | VIS – ES AP | 87.5 ± 10.5 | 85.5 ± 9.6 | 0.118 | 0.571 |
|  | VIS - ES ML | 89.9 ± 4.86 | 87.7 ± 5.75 | 0.002 | 0.623 |
|  | VIS - Jerk AP acc | 1.78 ± 0.49 | 2.04 ± 0.61 | 0.001 | 0.639 |
|  | VIS - Jerk ML acc | 1.81 ± 0.51 | 2.01 ± 0.61 | 0.016 | 0.597 |
|  | VIS – Jerk AP AngVel | 1.376 ± 0.307 | 1.429 ± 0.344 | 0.117 | 0.538 |
|  | VIS – Jerk ML AngVel | 1.329 ± 0.356 | 1.409 ± 0.352 | 0.029 | 0.586 |
|  | VIS – PSD AngVal AP | 7.685 ± 5.845 | 9.258 ± 7.677 | 0.022 | 0.578 |
|  | VIS – PSD AngVal ML | 3.131 ± 1.887 | 3.869 ± 3.869 | 0.025 | 0.542 |
|  | VES – Acceleration AP RMS | 2.418 ± 1.510 | 2.378 ± 0.997 | 0.765 | 0.511 |
|  | VES – Acceleration ML RMS | 4.522 ± 1.962 | 4.442 ± 1.653 | 0.672 | 0.513 |
|  | VES – AngVel AP RMS | 1.770 ± 0.599 | 1.831 ± 0.606 | 0.321 | 0.544 |
|  | VES – AngVel ML RMS | 1.580 ± 1.240 | 1.574 ± 0.669 | 0.953 | 0.543 |
|  | VES – ES AP | 0.696 ± 0.217 | 0.711 ± 0.154 | 0.474 | 0.515 |
|  | VES – ES ML | 0.812 ± 0.120 | 0.807 ± 0.097 | 0.625 | 0.546 |
|  | VES - Jerk AP acc. | 2.78 ± 1.24 | 3.23 ± 2.04 | 0.063 | 0.588 |
|  | VES – Jerk ML acc. | 2.795 ± 1.969 | 3.170 ± 3.906 | 0.214 | 0.550 |
|  | VES – Jerk AP AngVel | 1.893 ± 0.987 | 2.008 ± 0.987 | 0.377 | 0.518 |
|  | VES – Jerk ML AngVel | 1.787 ± 0.894 | 1.906 ± 1.209 | 0.264 | 0.548 |
|  | VES – PSD AngVal AP | 16.555 ± 18.444 | 18.397 ± 21.970 | 0.369 | 0.542 |
|  | VES – PSD AngVal ML | 12.685 ± 86.712 | 10.394 ± 34.636 | 0.730 | 0.522 |
|  | SOM – Acceleration AP RMS | 1.182 ± 0.415 | 1.203 ± 0.386 | 0.623 | 0.518 |
|  | SOM – Acceleration ML RMS | 1.120 ± 0.272 | 1.102 ± 0.403 | 0.592 | 0.551 |
|  | SOM – AngVel AP RMS | 1.013 ± 0.041 | 1.023 ± 0.117 | 0.242 | 0.508 |
|  | SOM – AngVel ML RMS | 1.058 ± 0.080 | 1.063 ± 0.088 | 0.568 | 0.503 |
|  | SOM – ES AP | 97.0 ± 7.6 | 96.5 ± 6.8 | 0.461 | 0.528 |
|  | SOM – ES ML | 99.3 ± 2.2 | 99.5 ± 2.3 | 0.575 | 0.524 |
|  | SOM – Jerk AP acc. | 1.138 ± 0.170 | 1.137 ± 0.195 | 0.977 | 0.527 |
|  | SOM – Jerk ML acc. | 1.088 ± 0.179 | 1.083 ± 0.241 | 0.798 | 0.533 |
|  | SOM – Jerk AP AngVel | 1.029 ± 0.083 | 1.037 ± 0.135 | 0.491 | 0.513 |
|  | SOM – Jerk ML AngVel | 1.048 ± 0.088 | 1.047 ± 0.102 | 0.851 | 0.515 |
|  | SOM – PSD AngVal AP | 1.131 ± 0.384 | 1.267 ± 1.711 | 0.320 | 0.505 |
|  | SOM – PSD AngVal ML | 1.565 ± 1.528 | 1.561 ± 1.076 | 0.978 | 0.506 |
| Limits of Stability (LOS) | LOS – AngVel AP MAX | 0.600 ± 0.259 | 0.560 ± 0.243 | 0.281 | 0.550 |
|  | LOS – AngVel ML MAX | 0.085 ± 0.034 | 0.083 ± 0.033 | 0.714 | 0.502 |
|  | LOS – JerkAngVel AP | 36.38 ± 14.62 | 34.64 ± 24.02 | 0.531 | 0.600 |
|  | LOS - Jerk AngVel ML | 26.24 ± 3.15 | 24.96 ± 2.79 | 0.004 | 0.643 |
| Sit to Stand Five Times (STS5) | STS5 - SiStSi Duration | 2.05 ± 0.43 | 2.44 ± 0.78 | <0.001 | 0.671 |
|  | STS5 - SiSt Duration | 0.97 ± 0.19 | 1.17 ± 0.39 | <0.001 | 0.685 |
|  | STS5 - StSi Duration | 1.08 ± 0.26 | 1.32 ± 0.55 | <0.001 | 0.651 |
|  | STS5 - SiStSi AngVel | 87.48 ± 20.89 | 76.71 ± 21.99 | 0.001 | 0.639 |
|  | STS5 - SiSt AngVel | 90.39 ± 20.53 | 79.22 ± 21.09 | <0.001 | 0.648 |
|  | STS5 - StSi AngVel | 84.19 ± 22.11 | 74.54 ± 22.79 | 0.003 | 0.621 |
|  | STS5 - SiStSi Jerk AngVel | 1592.92 ± 553.47 | 1341.82 ± 651.17 | 0.004 | 0.646 |
|  | STS5 - SiSt Jerk AngVel | 1696.09 ± 641.65 | 1367.70 ± 686.31 | 0.001 | 0.66 |
|  | STS5 - StSi Jerk AngVel | 1507.23 ± 519.93 | 1318.21 ± 650.68 | 0.025 | 0.625 |
| Timed Up and Go (TUG) | TUG - Acceleration AP RMS | 0.15 ± 0.03 | 0.13 ± 0.03 | 0.001 | 0.636 |
|  | TUG – Acceleration MLRMS | 0.119 ± 0.024 | 0.115 ± 0.024 | 0.205 | 0.553 |
|  | TUG - Acceleration V RMS | 0.14 ± 0.03 | 0.14 ± 0.03 | 0.048 | 0.577 |
|  | TUG - AngVel AP RMS | 58.57 ± 14.43 | 52.84 ± 13.51 | 0.005 | 0.604 |
|  | TUG - AngVel ML RMS | 135.31 ± 23.72 | 123.70 ± 23.48 | 0.001 | 0.636 |
|  | TUG - AngVel V RMS | 63.69 ± 9.39 | 56.85 ± 10.47 | <0.001 | 0.688 |
|  | TUG - Gait Velocity | 0.77 ± 0.11 | 0.68 ± 0.12 | <0.001 | 0.693 |
|  | TUG - Step Time | 1.04 ± 0.08 | 1.08 ± 0.13 | 0.042 | 0.567 |
|  | TUG – Step Length | 0.39 ± 0.04 | 0.36 ± 0.04 | <0.001 | 0.703 |
|  | TUG – Gait Symmetry | 4.501 ± 1.795 | 4.640 ± 2.626 | 0.660 | 0.507 |
|  | TUG - Turning Time | 2.11 ± 0.43 | 2.43 ± 0.61 | <0.001 | 0.665 |
|  | TUG - Turning AngVel RMS | 84.64 ± 10.33 | 76.63 ± 13.83 | <0.001 | 0.673 |
|  | TUG - Turning AngVel MAX | 113.78 ± 10.89 | 104.93 ± 16.31 | <0.001 | 0.659 |
| Motor Function (MF) | MF - Knee Extension Range | 4.29 ± 1.55 | 3.69 ± 1.82 | 0.013 | 0.624 |
|  | MF - Knee Flexion Range | 131.73 ± 13.38 | 124.95 ± 12.99 | <0.001 | 0.66 |
| Choice Reaction Test  (CRT) | Movement Time | 0.618 ± 0.151 | 0.591 ± 0.114 | 0.169 | 0.547 |
|  | Information Processing Speed | 7.09 ± 1.28 | 5.82 ± 1.27 | <0.001 | 0.753 |
| Falls Efficacy Scale (FES) | Fall Efficacy Scale Score | 9.73 ± 2.98 | 14.96 ± 4.92 | <0.001 | 0.834 |

Notations: EOFS: Eyes open on firm surface; ECFS: Eyes closed on firm surface; EOMS: Eyes open on mat (foam) surface; ECMS: Eyes closed on mat (foam) surface; VIS: Visual; VES: Vestibular; SOM: Somatosenosry; AP: anteroposterior; ML: Mediolateral; RMS: root mean square; ES: equilibrium score; AngVel: angular velocity; acc: acceleration; SiSt: sit to stand; StSi: stand to sit; SiStSi: Sit to stand then sit.

**Supplementary Table S2:** 38 Significant Outcome Measures from Seven Subtests

| **Subtests** | **Outcome Measures** | **Non-fallers** (mean ± SD) | **Fallers**  (mean ± SD) | **T test (p)** | **AUC** |
| --- | --- | --- | --- | --- | --- |
| Sensory Integration Test (SIT) | EOMS - Acceleration AP RMS | 0.01 ± 0.01 | 0.02 ± 0.01 | 0.033 | 0.583 |
|  | EOMS - Acceleration ML RMS | 0.006 ± 0.001 | 0.007 ± 0.002 | 0.002 | 0.618 |
|  | EOMS – AngVel AP RMS | 0.73 ± 0.13 | 0.77 ± 0.16 | 0.062* | 0.571 |
|  | EOMS - ES AP | 72.38 ± 7.11 | 70.49 ± 6.96 | 0.066* | 0.591 |
|  | EOMS - ES ML | 84.04 ± 4.34 | 82.03 ± 5.20 | 0.005 | 0.615 |
|  | EOFS - RMS ML acc | 0.0023 ± 0.001 | 0.0025 ± 0.001 | 0.090* | 0.569 |
|  | EOFS - Jerk AP acc | 0.043 ± 0.012 | 0.039 ± 0.009 | 0.009 | 0.592 |
|  | ECFS - Jerk AP acc | 0.049 ± 0.016 | 0.044 ± 0.012 | 0.028 | 0.574 |
|  | VIS - RMS AP ang | 1.38 ± 0.22 | 1.47 ± 0.29 | 0.033 | 0.581 |
|  | VIS - ES ML | 89.9 ± 4.86 | 87.7 ± 5.75 | 0.002 | 0.623 |
|  | VIS - Jerk AP acc | 1.78 ± 0.49 | 2.04 ± 0.61 | 0.001 | 0.639 |
|  | VIS - Jerk ML acc | 1.81 ± 0.51 | 2.01 ± 0.61 | 0.016 | 0.597 |
|  | VES - Jerk AP acc | 2.78 ± 1.24 | 3.23 ± 2.04 | 0.063* | 0.59 |
| Limits of Stability (LOS) | LOS - Jerk AngVel ML | 26.24 ± 3.15 | 24.96 ± 2.79 | 0.004 | 0.643 |
| Sit to Stand Five Times (STS5) | STS5 - SiStSi Duration | 2.05 ± 0.43 | 2.44 ± 0.78 | <0.001 | 0.671 |
|  | STS5 - SiSt Duration | 0.97 ± 0.19 | 1.17 ± 0.39 | <0.001 | 0.685 |
|  | STS5 - StSi Duration | 1.08 ± 0.26 | 1.32 ± 0.55 | <0.001 | 0.651 |
|  | STS5 - SiStSi AngVel | 87.48 ± 20.89 | 76.71 ± 21.99 | 0.001 | 0.639 |
|  | STS5 - SiSt AngVel | 90.39 ± 20.53 | 79.22 ± 21.09 | <0.001 | 0.648 |
|  | STS5 - StSi AngVel | 84.19 ± 22.11 | 74.54 ± 22.79 | 0.003 | 0.621 |
|  | STS5 - SiStSi Jerk AngVel | 1592.92 ± 553.47 | 1341.82 ± 651.17 | 0.004 | 0.646 |
|  | STS5 - SiSt Jerk AngVel | 1696.09 ± 641.65 | 1367.70 ± 686.31 | 0.001 | 0.66 |
|  | STS5 - StSi Jerk AngVel | 1507.23 ± 519.93 | 1318.21 ± 650.68 | 0.025 | 0.625 |
| Timed Up and Go (TUG) | TUG - Acceleration AP RMS | 0.15 ± 0.03 | 0.13 ± 0.03 | 0.001 | 0.636 |
|  | TUG - Acceleration V RMS | 0.14 ± 0.03 | 0.14 ± 0.03 | 0.048 | 0.577 |
|  | TUG - AngVel AP RMS | 58.57 ± 14.43 | 52.84 ± 13.51 | 0.005 | 0.604 |
|  | TUG - AngVel ML RMS | 135.31 ± 23.72 | 123.70 ± 23.48 | 0.001 | 0.636 |
|  | TUG - AngVel V RMS | 63.69 ± 9.39 | 56.85 ± 10.47 | <0.001 | 0.688 |
|  | TUG - Gait Velocity | 0.77 ± 0.11 | 0.68 ± 0.12 | <0.001 | 0.693 |
|  | TUG - Step Time | 1.04 ± 0.08 | 1.08 ± 0.13 | 0.042 | 0.567 |
|  | TUG - Step Length | 0.39 ± 0.04 | 0.36 ± 0.04 | <0.001 | 0.703 |
|  | TUG - Turning Time | 2.11 ± 0.43 | 2.43 ± 0.61 | <0.001 | 0.665 |
|  | TUG - Turning AngVel RMS | 84.64 ± 10.33 | 76.63 ± 13.83 | <0.001 | 0.673 |
|  | TUG - Turning AngVel MAX | 113.78 ± 10.89 | 104.93 ± 16.31 | <0.001 | 0.659 |
| Motor Function (MF) | MF - Knee Extension Range | 4.29 ± 1.55 | 3.69 ± 1.82 | 0.013 | 0.624 |
|  | MF - Knee Flexion Range | 131.73 ± 13.38 | 124.95 ± 12.99 | <0.001 | 0.66 |
| Choice Reaction Test  (CRT) | Information Processing Speed | 7.09 ± 1.28 | 5.82 ± 1.27 | <0.001 | 0.753 |
| Falls Efficacy Scale  (FES) | Fall Efficacy Scale Score | 9.73 ± 2.98 | 14.96 ± 4.92 | <0.001 | 0.834 |

* Indicate marginal significance since the p-value is in the range of 0.05˂p≤0.1

Notations: EOMS: Eyes open on mat (foam) surface; EOFS: Eyes open on firm surface; ECFS: Eyes closed on firm surface; VIS: Visual; VES: Vestibular; SOM: Somatosenosry; AP: anteroposterior; ML: Mediolateral; RMS: root mean square; ES: equilibrium score; AngVel: angular velocity; acc: acceleration; SiSt: sit to stand; StSi: stand to sit; SiStSi: Sit to stand then sit.
